# Supplementary material for: Empirical methods for controlling false positives and estimating confidence in ChIP-Seq peaks
Source: BMC Bioinformatics. 2008 Dec 5;9:523. doi: 10.1186/1471-2105-9-523 (PMC2628906; doi:10.1186/1471-2105-9-523)
Supplement: Additional File 1 — A variety of html documents from the USeq web site detailing the available applications, their best usage, output file type descriptions, command line menus, etc. [file 1471-2105-9-523-S1.zip › USeqUserGuides/JavaDoc.zip!/JavaDoc/bioroot/class-use/DBUtil.html]

Uses of Class bioroot.DBUtil


|  |  |  |
| --- | --- | --- |
| **Overview** \| **Package** \| **Class** \| **Use** \| **Tree** \| **Deprecated** \| **Index** \| **Help** | |  |
| PREV   NEXT | **FRAMES**    **NO FRAMES**     **All Classes** |  |


---


## **Uses of Class bioroot.DBUtil**

| Packages that use DBUtil | |
| --- | --- |
| **bioroot** |  |
| **bioroot.antibody** |  |
| **bioroot.oligo** |  |
| **bioroot.plasmid** |  |
| **bioroot.strain** |  |

Uses of DBUtil in bioroot

| Methods in bioroot that return DBUtil | |
| --- | --- |
| `DBUtil` | `UserRegistrationBean.getBioroot()` |

| Methods in bioroot with parameters of type DBUtil | |
| --- | --- |
| `static java.lang.String` | `DBUtil.buildSqlStatement(java.lang.String reagentType, UserBean user, DBUtil bioRoot, boolean descending)`             Creates a sql statement given a UserBean and a boolean as to whether to add a DESC to the order by where appropriate. |
| `boolean` | `ReagentBean.canView(UserBean user, DBUtil bioRoot)` |
| `boolean` | `LoginBean.checkParams(DBUtil bioRoot)` |
| `static java.lang.String` | `Search.checkPlasmidDBFile(DBUtil bioRoot)`             Looks for an upto date blast database file. |
| `UserBean` | `LoginBean.createUserBean(DBUtil bioRoot, java.lang.String host, java.lang.String IP)` |
| `static java.util.ArrayList` | `Archive.fetchFiles(PlasmidBean[] plasmids, StrainBean[] strains, AntibodyBean[] antibodies, java.lang.String filePath, DBUtil bioRoot)`             Fetches any plasmid or strain files. |
| `java.lang.String` | `ReagentBean.getLabGroupName(DBUtil bioRoot)` |
| `int` | `UserBean.getLabGroupSchedule(DBUtil bioRoot)` |
| `static LabGroup[]` | `CronArchive.getLabGroupsToArchive(DBUtil bioRoot)`             Identifies lab groups that should be archived based on their last archive date and schedule. |
| `java.lang.String` | `ReagentBean.getOrganismName(DBUtil bioRoot)` |
| `Organization` | `ReagentBean.getOrganization(DBUtil bioRoot)` |
| `java.lang.String` | `ReagentBean.getOwnerName(DBUtil bioRoot)` |
| `static java.sql.ResultSet` | `AccountManagement.getUser(DBUtil mysql, java.lang.String login)`             fetch all items from a specific user |
| `static java.lang.String[]` | `AccountManagement.getUsers(DBUtil bioroot, int labGroupId)`             Returns a String[] of Users belonging to a particular labgroup. |
| `java.lang.String[]` | `Preference.getVisibilities(DBUtil bioRoot, UserBean userBean)` |
| `static java.lang.String[]` | `Util.getVisibilities(UserBean userBean, DBUtil bioRoot)`             If user is part of an organization, it returns WWW, orgName, Lab Mates, Private. |
| `java.lang.String` | `ReagentBean.getVisibility(DBUtil bioRoot)` |
| `boolean` | `ReagentBean.isReagentNameUnique(DBUtil bioRoot, java.lang.String tableName)`             checks to see if oligo name is unique within labgroup, assumes a connection has been made |
| `void` | `ContactBean.loadContactInfo(DBUtil bioRoot)` |
| `void` | `Organization.loadOrganizationInfo(DBUtil bioRoot, int labGroupId)` |
| `static void` | `CronArchive.loadSuperUserEmailAddresses(DBUtil bioRoot, LabGroup[] labGroups)`             Loads an array of LabGroup[] with superuser email addresses. |
| `static AntibodyBean[]` | `Archive.makeAntibodyBeans(DBUtil bioRoot, UserBean userBean)`             Makes an array of all visibile AntibodyBeans |
| `static java.io.File` | `CronArchive.makeArchive(DBUtil bioRoot, UserBean superUser)`             Returns null or a zip file containing a labGroup's full archive. |
| `static java.lang.String` | `Search.makeMultiFASTA(java.lang.String tableName, DBUtil bioRoot)` |
| `static OligoBean[]` | `Archive.makeOligoBeans(DBUtil bioRoot, UserBean userBean)`             Makes an array of all visibile OligoBeans |
| `static PlasmidBean[]` | `Archive.makePlasmidBeans(DBUtil bioRoot, UserBean userBean)`             Makes an array of all visibile PlasmidBeans |
| `static StrainBean[]` | `Archive.makeStrainBeans(DBUtil bioRoot, UserBean userBean)`             Makes an array of all visibile StrainBeans |
| `SearchHit[]` | `SearchBean.searchForSimilarMatches(java.lang.String[] columns, java.lang.String[] words, DBUtil util, boolean searchForWordBoundaryMatches, UserBean userBean)`             Searches a list of columns for a set of words, case insensitive, %word% matches. |
| `void` | `UserRegistrationBean.setBioroot(DBUtil util)` |
| `static boolean` | `CronArchive.setLastArchiveDate(LabGroup[] labGroups, DBUtil bioRoot)`             If archived will set that labgroups lastArchive to today |
| `boolean` | `OrganismBean.submitNew(DBUtil bioRoot, UserBean user)` |
| `boolean` | `MarkerBean.submitNew(DBUtil bioRoot, UserBean user)` |
| `boolean` | `GeneBean.submitNew(DBUtil bioRoot, UserBean user)` |
| `boolean` | `CellTypeBean.submitNew(DBUtil bioRoot, UserBean user)` |
| `boolean` | `ReagentBean.updateCommonAccessFields(DBUtil bioRoot, java.lang.String reagentType)`             Just updates fields that are accessible by all lab group memebers, lastUser, comments given a reagent type: Oligo or Plasmid. |
| `boolean` | `OrganismBean.updateOld(DBUtil bioRoot, UserBean user)` |
| `boolean` | `MarkerBean.updateOld(DBUtil bioRoot, UserBean user)` |
| `boolean` | `GeneBean.updateOld(DBUtil bioRoot, UserBean user)` |
| `boolean` | `CellTypeBean.updateOld(DBUtil bioRoot, UserBean user)` |

Constructors in bioroot with parameters of type DBUtil

`CellTypeBean(int id,
DBUtil bioRoot)`

`GeneBean(int id,
DBUtil bioRoot)`

`MarkerBean(int id,
DBUtil bioRoot)`

`OrganismBean(int id,
DBUtil bioRoot)`

`Organization(DBUtil bioRoot,
int labGroupId)`

`UserBean(DBUtil db,
LoginBean loginBean)`

Uses of DBUtil in bioroot.antibody

| Methods in bioroot.antibody with parameters of type DBUtil | |
| --- | --- |
| `static void` | `NewAntibody.addAnAntibodyPrep(AntibodyBean bean, DBUtil bioRoot)`             Adds a new antibodyPrep to the AntibodyBean |
| `static void` | `AntibodyBase.addPlasmid(AntibodyBean bean, java.util.HashMap nameValue, DBUtil bioRoot, UserBean userBean)` |
| `static void` | `AntibodyBase.addStrain(AntibodyBean bean, java.util.HashMap nameValue, DBUtil bioRoot, UserBean userBean)` |
| `static void` | `NewAntibody.addUseDilution(AntibodyBean bean, DBUtil bioRoot, int idAntibodyPrep)`             Adds a new UseDilution to the indicated AntibodyPrep |
| `static int` | `AntibodySpreadSheet.addUseDilutionBlankLines(boolean useDilutionsPresent, java.lang.String[] items, AntibodyPrep prep, int k, DBUtil bioRoot)`             Adds "" to items based on the number of use dilutions present in the Antibody Prep bean. |
| `static java.lang.String` | `AntibodyModify.deleteAntibody(AntibodyBean ab, UserBean userBean, DBUtil bioRoot)`             Attempts to delete an antibody, if sucessful, returns a "" String, otherwise an error message. |
| `static int` | `AntibodyBase.deleteAntibodyPrepIndex(AntibodyBean a, DBUtil bioRoot, java.util.HashMap m)`             Scans a hashmap for an antibody prep to delete returns the index number |
| `static java.lang.String` | `AntibodyBase.deleteUseDilutionIndex(AntibodyBean a, DBUtil bioRoot, java.util.HashMap m)`             Scans a hashmap for a use dilution to delete returns the index string or null. |
| `static AntibodyBean[]` | `AntibodySpreadSheet.fetchAntibodyBeans(java.lang.String sql, DBUtil bioRoot)`             Build AntibodyBean[] from Antibody table in database. |
| `static java.lang.String` | `AntibodySpreadSheet.fetchAntibodyTableRows(UserBean userBean, AntibodyBean[] beans, int[] startStop, DBUtil bioRoot, boolean checked, boolean allTabs)`             Returns table rows for every oligo according to a Preference, if allTabs==true, then all features are returned in a tab delimited format, otherwise its HTML. |
| `static java.lang.String` | `AntibodyBase.getAntibodyForm(AntibodyBean ab, DBUtil bioRoot, UserBean userBean)`             Returns an antibody form filled with the values in the bean. |
| `static void` | `AntibodyBase.getAntibodyPrepForm(AntibodyPrep a, DBUtil bioRoot, java.lang.StringBuffer b, int index)` |
| `static void` | `AntibodyBase.getAntibodyPrepForms(AntibodyBean ab, DBUtil bioRoot, java.lang.StringBuffer b)` |
| `static void` | `AntibodyBase.getAntibodyPrepReport(AntibodyPrep a, DBUtil bioRoot, java.lang.StringBuffer b, int index, boolean commonForm)` |
| `static void` | `AntibodyBase.getAntibodyPrepReports(AntibodyBean ab, DBUtil bioRoot, java.lang.StringBuffer b, boolean commonForm)` |
| `AntibodyPrep[]` | `AntibodyBean.getAntibodyPreps(DBUtil bioRoot)` |
| `static java.lang.String` | `AntibodyBase.getAntibodyReport(AntibodyBean ab, DBUtil bioRoot, UserBean user, boolean commonForm)`             Returns an antibody report filled with the values in the bean. |
| `java.lang.String` | `AntibodyBean.getGeneId(DBUtil bioRoot)` |
| `java.lang.String` | `AntibodyBean.getGeneName(DBUtil bioRoot)` |
| `java.lang.String` | `AntibodyBean.getHotLinkedPlasmids(DBUtil bioRoot)`             Returns HTML plasmid names for display. |
| `java.lang.String` | `AntibodyBean.getHotLinkedStrains(DBUtil bioRoot)`             Returns HTML strain names for display. |
| `java.lang.String` | `AntibodyPrep.getOrganismName(DBUtil bioRoot)` |
| `java.lang.String[]` | `AntibodyBean.getPlasmidConcats(DBUtil bioRoot)`             Returns an array of id: plasmidName, or empty String[]{""} if there are none. |
| `java.lang.String[]` | `AntibodyBean.getStrainConcats(DBUtil bioRoot)`             Returns an array of id: strainName, or empty String[]{""} if there are none. |
| `static void` | `AntibodyBase.getUseDilutionForms(AntibodyPrep a, DBUtil bioRoot, java.lang.StringBuffer b, int apbIndex)`             Pumps out useDilution forms, should always have one with each Prep |
| `static void` | `AntibodyBase.getUseDilutionReports(AntibodyPrep a, DBUtil bioRoot, java.lang.StringBuffer b, int apbIndex, boolean commonForm)`             Pumps out useDilution reports |
| `UseDilution[]` | `AntibodyPrep.getUseDilutions(DBUtil bioRoot)` |
| `boolean` | `AntibodyBean.isAntibodyNameUnique(DBUtil bioRoot)`             checks to see if antibody name is unique within labgroup, assumes a connection has been made |
| `static void` | `NewAntibody.loadAntibodyBean(java.util.HashMap nameValue, DBUtil bioRoot, AntibodyBean ab, javax.servlet.http.HttpServletRequest request)` |
| `static void` | `NewAntibody.loadAntibodyPrepBeans(java.util.HashMap nameValue, DBUtil bioRoot, AntibodyBean ab, javax.servlet.http.HttpServletRequest request)` |
| `static void` | `AntibodySpreadSheet.makeAndSetAntibodyBeans(javax.servlet.http.HttpSession session, DBUtil bioRoot, UserBean userBean)`             Makes an array of AntibodyBeans based on Users AntibodyPreferences and sets them in the session. |
| `static int` | `AntibodyBase.newUseDilutionIndex(AntibodyBean a, DBUtil bioRoot, java.util.HashMap m)`             Scans a hashmap for a newUseDilution selection, returns the index number |
| `static AntibodyBean[]` | `AntibodySpreadSheet.orderAntibodies(java.lang.String featureName, UserBean userBean, AntibodyBean[] beans, DBUtil bioRoot)`             Orders antibodys, sort of nasty, can't figure another way, reflection is way too slow. |
| `static void` | `AntibodyBase.removePlasmid(AntibodyBean bean, java.util.HashMap nameValue, DBUtil bioRoot)` |
| `static void` | `AntibodyBase.removeStrain(AntibodyBean bean, java.util.HashMap nameValue, DBUtil bioRoot)` |
| `boolean` | `AntibodyBean.saveNewAntibodyPreps(DBUtil bioRoot)` |
| `boolean` | `AntibodyPrep.saveNewUseDilutions(DBUtil bioRoot)` |
| `boolean` | `AntibodyBean.savePlasmidIds(DBUtil bioRoot, boolean deleteOld)`             Can delete old plasmidIds from AntibodyPlasmid table, and add new entries if any are present in the plasmidConcats. |
| `boolean` | `AntibodyBean.saveStrainIds(DBUtil bioRoot, boolean deleteOld)`             Can delete old strainIds from AntibodyStrain table, and add new entries if any are present in the strainConcats. |
| `boolean` | `AntibodyPrep.submitNew(DBUtil bioRoot)`             Makes a new AntibodyPrep table entry. |
| `boolean` | `AntibodyBean.submitNew(DBUtil bioRoot)`             Makes a new Antibody table entry, don't forget to save associated strains and plasmids after updating this bean with its new id number. |
| `boolean` | `UseDilution.submitNew(DBUtil bioRoot, int antibodyPrepId)` |
| `static java.lang.String` | `AntibodyModify.updateAntibody(AntibodyBean bean, UserBean userBean, DBUtil bioRoot)`             Attempts to update a AntibodyBean in the database, only checks that the name is unique if it has changed. |
| `static java.lang.String` | `AntibodyModify.updateAntibodyPreps(AntibodyBean bean, DBUtil bioRoot)`             Attempts to update the AntibodyPreps and UseDilutions in the database. |
| `static boolean` | `AntibodyModify.updateCommonAccessFields(AntibodyPrep[] preps, DBUtil bioRoot)`             Just update the commonly accessible fields in each antibodyPrep as well as the useDilutions. |
| `boolean` | `AntibodyPrep.updateCommonAccessFields(DBUtil bioRoot)`             Just updates fields that are accessible by all lab group memebers. |
| `boolean` | `UseDilution.updateOld(DBUtil bioRoot)` |
| `boolean` | `AntibodyPrep.updateOld(DBUtil bioRoot)`             Updates a Antibody prep entry |
| `boolean` | `AntibodyBean.updateOld(DBUtil bioRoot, UserBean submitter)`             Updates a Antibody entry, don't forget to update geneId |

Constructors in bioroot.antibody with parameters of type DBUtil

`AntibodyBean(int id,
DBUtil bioRoot)`
  
          Assumes id is not zero

Uses of DBUtil in bioroot.oligo

| Methods in bioroot.oligo with parameters of type DBUtil | |
| --- | --- |
| `static java.lang.String` | `OligoModify.deleteOligo(OligoBean oligoBean, UserBean userBean, DBUtil bioRoot)`             Attempts to delete an oligo, if sucessful, returns a "" String, otherwise an error message. |
| `static OligoBean[]` | `OligoSpreadSheet.fetchOligoBeans(java.lang.String sql, DBUtil bioRoot)`             Build OligoBean[] from Oligo table in database. |
| `static java.lang.String` | `OligoSpreadSheet.fetchOligoTableRows(Preference pref, OligoBean[] beans, int[] startStop, DBUtil bioRoot, boolean checked, boolean allTabs)`             Returns table rows for every oligo according to an OligoPreference, if allTabs==true, then all features are returned in a tab delimited format, otherwise its HTML. |
| `java.lang.String` | `OligoBean.getGeneName(DBUtil bioRoot)` |
| `static java.lang.String` | `OligoBase.getOligoForm(OligoBean ob, DBUtil bioRoot, UserBean userBean)`             Returns an oligo form filled it with the values in the oligo bean. |
| `static java.lang.String` | `OligoBase.getOligoReport(OligoBean ob, DBUtil bioRoot, UserBean userBean, boolean commonForm)`             Returns an oligo report. |
| `boolean` | `OligoBean.isOligoNameUnique(DBUtil bioRoot)`             checks to see if oligo name is unique within labgroup, assumes a connection has been made |
| `static void` | `OligoSpreadSheet.makeAndSetOligoBeans(javax.servlet.http.HttpSession session, DBUtil bioRoot, UserBean userBean)`             Makes an array of OligoBeans based on Users OligoPreferences and sets them in the session. |
| `static OligoBean[]` | `OligoSpreadSheet.orderOligos(java.lang.String featureName, Preference pref, OligoBean[] beans, DBUtil bioRoot)`             Orders oligos, sort of nasty, can't figure another way, reflection is way too slow. |
| `boolean` | `OligoBean.submitNew(DBUtil bioRoot)` |
| `boolean` | `OligoBean.updateOld(DBUtil bioRoot, UserBean submitter)` |
| `static java.lang.String` | `OligoModify.updateOligo(OligoBean oligoBean, UserBean userBean, javax.servlet.http.HttpServletRequest request, DBUtil bioRoot)`             Attempts to update an OligoBean in the database, only checks that the name is unique if it has changed. |

Constructors in bioroot.oligo with parameters of type DBUtil

`OligoBean(int id,
DBUtil bioRoot)`

`OligoBean(java.lang.String[] cells,
IdNameMatch[] genes,
IdNameMatch[] organisms,
UserBean userBean,
DBUtil bioRoot)`
  
          For bulk file upload should be 17? cells
Name, Alias Oligo Sequence, Matching Gene Sequence, uM Stock Concentration, ng/uL Stock Concentration, Volume(uL), Purification,
0 1 2 3 4 5 6 7
Modifications, Orientation, Type, Storage Location, Availibility, VisibilityBean, Handling Instructions, Target Gene Name, Organism, Notes
8 9 10 11 12 13 14 15 16 17

Uses of DBUtil in bioroot.plasmid

| Methods in bioroot.plasmid with parameters of type DBUtil | |
| --- | --- |
| `static void` | `NewPlasmid.addStrain(PlasmidBean bean, java.util.HashMap nameValue, int labGroupId, DBUtil bioRoot)` |
| `static java.lang.String` | `PlasmidModify.deletePlasmid(PlasmidBean plasmidBean, UserBean userBean, DBUtil bioRoot)`             Attempts to delete an plasmid, if sucessful, returns a "" String, otherwise an error message. |
| `static PlasmidBean[]` | `PlasmidSpreadSheet.fetchPlasmidBeans(java.lang.String sql, DBUtil bioRoot)`             Build PlasmidBean[] from Plasmid table in database. |
| `static java.lang.String` | `PlasmidSpreadSheet.fetchPlasmidTableRows(UserBean userBean, PlasmidBean[] beans, int[] startStop, DBUtil bioRoot, boolean checked, boolean allTabs)`             Returns table rows for every oligo according to a Preference, if allTabs==true, then all features are returned in a tab delimited format, otherwise its HTML. |
| `static void` | `PlasmidBase.findOligoMatches(UserBean user, PlasmidBean[] plasmids, DBUtil bioRoot)`             Uses Blast to find oligo matches to a given array of plasmid from all the oligos in a labgroup visible to the user. |
| `static void` | `PlasmidBase.findOligos(UserBean user, PlasmidBean[] plasmids, DBUtil bioRoot)`             Uses Blast to find oligo matches to a given array of plasmid from all the oligos in a labgroup visible to the user. |
| `static void` | `PlasmidBase.findOligos(UserBean user, PlasmidBean plasmid, DBUtil bioRoot)`             Uses Blast to find oligo matches to a plasmid from all the oligos in a labgroup visible to the user. |
| `java.lang.String` | `PlasmidBean.getGeneName(DBUtil bioRoot)` |
| `java.lang.String` | `PlasmidBean.getHotLinkedStrains(DBUtil bioRoot)`             Returns HTML strain names for display. |
| `java.lang.String[]` | `PlasmidBean.getMarkers(DBUtil bioRoot)` |
| `static java.lang.String` | `PlasmidBase.getPlasmidForm(PlasmidBean ob, DBUtil bioRoot, UserBean userBean)`             Returns a plasmid form filled with the values in the bean. |
| `static java.lang.String` | `PlasmidBase.getPlasmidReport(PlasmidBean pb, DBUtil bioRoot, UserBean userBean, boolean commonForm)`             Returns an plasmid report. |
| `java.lang.String[]` | `PlasmidBean.getStrainConcats(DBUtil bioRoot)`             Returns an array of id: strainName, or empty String[]{""} if there are none. |
| `boolean` | `PlasmidBean.isPlasmidNameUnique(DBUtil bioRoot)`             checks to see if plasmid name is unique within labgroup, assumes a connection has been made |
| `static void` | `PlasmidSpreadSheet.makeAndSetPlasmidBeans(javax.servlet.http.HttpSession session, DBUtil bioRoot, UserBean userBean)`             Makes an array of PlasmidBeans based on Users PlasmidPreferences and sets them in the session. |
| `static PlasmidBean[]` | `PlasmidSpreadSheet.orderPlasmids(java.lang.String featureName, UserBean userBean, PlasmidBean[] beans, DBUtil bioRoot)`             Orders plasmids, sort of nasty, can't figure another way, reflection is way too slow. |
| `static void` | `NewPlasmid.removeStrain(java.util.HashMap nameValue, PlasmidBean bean, DBUtil bioRoot)` |
| `boolean` | `PlasmidBean.saveMarkers(DBUtil bioRoot, boolean deleteOld)` |
| `boolean` | `PlasmidBean.saveStrainIds(DBUtil bioRoot, boolean deleteOld)`             Can delete old strainIds from PlasmidStrain table, and add new entries if any are present in the strainConcats. |
| `boolean` | `PlasmidBean.submitNew(DBUtil bioRoot)` |
| `boolean` | `PlasmidBean.updateOld(DBUtil bioRoot, UserBean submitter)` |
| `static java.lang.String` | `PlasmidModify.updatePlasmid(PlasmidBean bean, UserBean userBean, java.util.HashMap nameValue, javax.servlet.http.HttpServletRequest request, DBUtil bioRoot)`             Attempts to update a PlasmidBean in the database, only checks that the name is unique if it has changed. |

Constructors in bioroot.plasmid with parameters of type DBUtil

`PlasmidBean(int id,
DBUtil bioRoot)`

`PlasmidBean(java.lang.String[] cells,
IdNameMatch[] genes,
IdNameMatch[] organisms,
IdNameMatch[] markerIDMatch,
UserBean userBean,
DBUtil bioRoot)`
  
          For bulk file upload should be 16 cells
0 1 2 3 4 5 6 7 8 9 10 11 12 13 14 15 16
Name Alias Source Recipients Reference Gene Organism Markers StrainIds Location Concentration Volume Purification Availability VisibilityBean Notes Sequence

Uses of DBUtil in bioroot.strain

| Methods in bioroot.strain with parameters of type DBUtil | |
| --- | --- |
| `static void` | `NewStrain.addParent(StrainBean bean, java.util.HashMap nameValue, DBUtil bioRoot, UserBean userBean)` |
| `static void` | `NewStrain.addPlasmid(StrainBean bean, java.util.HashMap nameValue, DBUtil bioRoot, UserBean userBean)` |
| `static java.lang.String` | `StrainModify.deleteStrain(StrainBean strainBean, UserBean userBean, DBUtil bioRoot)`             Attempts to delete an strain, if sucessful, returns a "" String, otherwise an error message. |
| `static StrainBean[]` | `StrainSpreadSheet.fetchStrainBeans(java.lang.String sql, DBUtil bioRoot)`             Build StrainBean[] from Strain table in database. |
| `static java.lang.String` | `StrainSpreadSheet.fetchStrainTableRows(UserBean userBean, StrainBean[] beans, int[] startStop, DBUtil bioRoot, boolean checked, boolean allTabs)`             Returns table rows for every oligo according to a Preference, if allTabs==true, then all features are returned in a tab delimited format, otherwise its HTML. |
| `java.lang.String` | `StrainBean.getCellTypeName(DBUtil bioRoot)`             Will look one time for a name, all other times will just return "". |
| `java.lang.String` | `StrainBean.getHotLinkedParents(DBUtil bioRoot)`             Returns HTML parent/ strain names for display. |
| `java.lang.String` | `StrainBean.getHotLinkedPlasmids(DBUtil bioRoot)`             Returns HTML plasmid names for display. |
| `java.lang.String[]` | `StrainBean.getMarkers(DBUtil bioRoot)` |
| `java.lang.String[]` | `StrainBean.getParentConcats(DBUtil bioRoot)`             Returns an array of id: strainName, or empty String[]{""} if there are none. |
| `java.lang.String[]` | `StrainBean.getPlasmidConcats(DBUtil bioRoot)`             Returns an array of id: plasmidName, or empty String[]{""} if there are none. |
| `static java.lang.String` | `StrainBase.getStrainForm(StrainBean sb, DBUtil bioRoot, UserBean userBean)`             Returns a strain form filled with the values in the bean. |
| `static java.lang.String` | `StrainBase.getStrainReport(StrainBean sb, DBUtil bioRoot, UserBean userBean, boolean commonForm)`             Returns an strain report. |
| `boolean` | `StrainBean.isStrainNameUnique(DBUtil bioRoot)`             checks to see if strain name is unique within labgroup, assumes a connection has been made |
| `static void` | `StrainSpreadSheet.makeAndSetStrainBeans(javax.servlet.http.HttpSession session, DBUtil bioRoot, UserBean userBean)`             Makes an array of StrainBeans based on Users StrainPreferences and sets them in the session. |
| `static StrainBean[]` | `StrainSpreadSheet.orderStrains(java.lang.String featureName, UserBean userBean, StrainBean[] beans, DBUtil bioRoot)`             Orders strains, sort of nasty, can't figure another way, reflection is way too slow. |
| `static void` | `NewStrain.removeParent(StrainBean bean, java.util.HashMap nameValue, DBUtil bioRoot)` |
| `static void` | `NewStrain.removePlasmid(StrainBean bean, java.util.HashMap nameValue, DBUtil bioRoot)` |
| `boolean` | `StrainBean.saveMarkers(DBUtil bioRoot, boolean deleteOld)` |
| `boolean` | `StrainBean.saveParentIds(DBUtil bioRoot, boolean deleteOld)`             Can delete old strainIds from StrainParent table, and add new entries if any are present in the parentConcats. |
| `boolean` | `StrainBean.savePlasmidIds(DBUtil bioRoot, boolean deleteOld)`             Can delete old plasmidIds from StrainPlasmid table, and add new entries if any are present in the plasmidConcats. |
| `boolean` | `StrainBean.submitNew(DBUtil bioRoot)`             Makes a new Strain table entry, don't forget to save associated markers and plasmids after updating this bean with its new id number. |
| `boolean` | `StrainBean.updateOld(DBUtil bioRoot, UserBean submitter)`             Updates a Strain entry, don't forget to update markers and plasmids. |
| `static java.lang.String` | `StrainModify.updateStrain(StrainBean bean, UserBean userBean, java.util.HashMap nameValue, javax.servlet.http.HttpServletRequest request, DBUtil bioRoot)`             Attempts to update a StrainBean in the database, only checks that the name is unique if it has changed. |
| `boolean` | `StrainBean.updateStrainCommonAccessFields(DBUtil bioRoot)`             Just updates fields that are accessible by all lab group memebers. |
| `static boolean` | `StrainModify.updateStrainCommonAccessFields(StrainBean bean, UserBean userBean, java.util.HashMap nameValue, DBUtil bioRoot)`             Just update the commonly accessible fields: restock, lastUser, remainingAliquots, comments |

Constructors in bioroot.strain with parameters of type DBUtil

`StrainBean(int id,
DBUtil bioRoot)`

`StrainBean(java.lang.String[] cells,
IdNameMatch[] cellTypes,
IdNameMatch[] organisms,
IdNameMatch[] markerIDMatch,
UserBean userBean,
DBUtil bioRoot)`
  
          For bulk file upload should be 23 cells
0 Name
1 Alias
2 Source
4 Reference
5 Type Bacteria, Yeast, Worm, Insect, Plant, Fish, Cell Line, Bird, Mammal or Other
6 Recipients
7 Organism name
8 Markers names, comma seperated
9 Plasmid Ids
10 Parent Strain Ids
11 Storage Location
12 Mutagen EMS, ENU, MMS, MNU, X-ray, UV, Transposon, T-DNA, Spontaneous or Other
13 Genotype/ Background
14 Phenotype
15 Growth Conditions
16 Restock
17 Remaining Aliquots
18 Array Type Extra Chromosomal or Integration
19 Seed Type Individual Line, Individual Pool, Set of Lines, or Set of Pools
20 Cell Type (Cell Lines)
21 Passage Number ( Cell Lines)
22 Mating Type ( Yeast) diploid, a, alpha, h+, or h-)
23 Availability
24 VisibilityBean
25 Notes

---


|  |  |  |
| --- | --- | --- |
| **Overview** \| **Package** \| **Class** \| **Use** \| **Tree** \| **Deprecated** \| **Index** \| **Help** | |  |
| PREV   NEXT | **FRAMES**    **NO FRAMES**     **All Classes** |  |


---
